# Supplementary material for: Insights into the Structure of Rubisco from Dinoflagellates-In Silico Studies
Source: Int J Mol Sci. 2021 Aug 7;22(16):8524. doi: 10.3390/ijms22168524 (PMC8395205; doi:10.3390/ijms22168524)
Supplement: Supplementary file 1 [file ijms-22-08524-s001.zip › ijms-1290571-supplementary.pdf]

Supplementary information

Insights into the structure of Rubisco from Dinoflagellates-*in silico* studies

Małgorzata Rydzy, Michał Tracz, Andrzej Szczepaniak, Joanna Grzyb*

Department of Biophysics, Faculty of Biotechnology, University of Wrocław, F. Joliot-Curie 14a str., 50-383 Wrocław, Poland

*joanna.grzyb@uwr.edu.pl

FormII

R. palustris	49	54	112	165	167	169	192	194	288	322	330	369	394
	E	T	N	I	K	K	K	DE	HR	H	KM	SGG	GGG
R. rubrum	49	54	112	165	167	169	192	194	288	322	330	369	394
	E	T	N	I	K	K	K	DE	HR	H	KM	SGG	GGG
Symbiodinium sp.	48	53	111	164	166	168	191	193	287	321	329	368	393
	E	T	N	I	K	K	K	DE	HR	H	KM	SGG	GGG

Figure S1: Comparison of amino acid identities in active site regions of Rubisco from *R. palustris, R. rubrum* and *Symbiodinium* sp.

Table S1. The comparison of the binding energies and its electrostatic component, calculated with Foldxsuite for studied protein structures.

	**Interface between monomers in dimer**			**Interface between dimers**		
RbcL origin	**IM**[‡]	**Binding energy** [kcal/mol]	**Electrostatic interactions** [kcal/mol]	**IM**	**Binding energy** [kcal/mol]	**Electrostatic interactions** [kcal/mol]
R. rubrum WT	**AB**	-50.90	-9.74	not applicable		
Symbiodinium sp., model (template: *R. rubrum*)	**AB**	-34.07	-7.49	not applicable		
Δloop[§] *R. rubrum* model (template: *R. rubrum)*	**AB**	-42.73	-8.43	not applicable		
Δloop[¶] *Symbiodinium* sp., model (template: *R. rubrum*)	**AB**	-34.07	-7.49	not applicable		
R. palustris	**AB**	-39.24	-13.44	**BC**	-4.66	-4.06
	**CD**	-41.09	-12.90	**DE**	-4.60	-4.12
	**EF**	-38.75	-13.27	**AF**	-8.31	-4.29
	$\overline{IM}$[#]	***-39.69***	***-13.20***	$\overline{IM}$	***-5.86***	***-4.16***
Symbiodinium sp., model (template: *R. palustris*)	**AB**	-47.55	-12.81	**BC**	1.44	-3.75
	**CD**	-46.98	-12.29	**DE**	-1.56	-3.29
	**EF**	-52.26	-12.03	**AF**	-3.32	-3.18
	$\overline{IM}$	***-48.93***	***-12.38***	$\overline{IM}$	***-1.15***	***-3.41***
Δloop *Symbiodinium* sp., model (template: *R. palustris*)	**AB**	-47.55	-12.81	**BC**	1.44	-3.75
	**CD**	-46.98	-12.29	**DE**	-1.56	-3.29
	**EF**	-52.26	-12.03	**AF**	-3.32	-3.18
	$\overline{IM}$	***-48.93***	***-12.38***	$\overline{IM}$	**-1.15**	**-3.41**
Δloop *R. rubrum* model (template: *R. palustris)*	**AB**	-61.99	-15.49	**BC**	8.81	-1.06
	**CD**	-46.93	-14.06	**DE**	11.06	0.50
	**EF**	-62.37	-14.89	**AF**	11.60	-0.53
	$\overline{IM}$	***-57.10***	***-14.81***	$\overline{IM}$	***10.49***	***-0.36***
R. rubrum WT model (template: R. palustris)	**AB**	-63.31	-15.49	**BC**	7.50	-1.21
	**CD**	-51.16	-14.14	**DE**	11.02	0.39
	**EF**	-58.62	-14.97	**AF**	9.71	-0.68
	$\overline{IM}$	***-57.70***	***-14.87***	$\overline{IM}$	***9.41***	***-0.50***

[‡]IM - interacting monomers

[§]the *R. rubrum* with a *Symbiodinium* sp. loop (insert 424) structure model

[¶]the *Symbiodinium* sp. without a loop (insert 424) structure model

[#]the arithmetic average of individual IM entries energy
